# Satellite Angular Velocity Estimation Based on Star Images and Optical Flow Techniques

**DOI:** 10.3390/s131012771

**Published:** 2013-09-25

**Authors:** Giancarmine Fasano, Giancarlo Rufino, Domenico Accardo, Michele Grassi

**Affiliations:** Department of Industrial Engineering (DII), University of Naples “Federico II”, P.le Tecchio 80, Naples I80125, Italy; E-Mails: giancarlo.rufino@unina.it (G.R.); domenico.accardo@unina.it (D.A.); michele.grassi@unina.it (M.G.)

**Keywords:** spacecraft angular velocity estimation, star field images, optical flow, performance analysis, hardware-in-the-loop simulation

## Abstract

An optical flow-based technique is proposed to estimate spacecraft angular velocity based on sequences of star-field images. It does not require star identification and can be thus used to also deliver angular rate information when attitude determination is not possible, as during platform de tumbling or slewing. Region-based optical flow calculation is carried out on successive star images preprocessed to remove background. Sensor calibration parameters, Poisson equation, and a least-squares method are then used to estimate the angular velocity vector components in the sensor rotating frame. A theoretical error budget is developed to estimate the expected angular rate accuracy as a function of camera parameters and star distribution in the field of view. The effectiveness of the proposed technique is tested by using star field scenes generated by a hardware-in-the-loop testing facility and acquired by a commercial-off-the shelf camera sensor. Simulated cases comprise rotations at different rates. Experimental results are presented which are consistent with theoretical estimates. In particular, very accurate angular velocity estimates are generated at lower slew rates, while in all cases the achievable accuracy in the estimation of the angular velocity component along boresight is about one order of magnitude worse than the other two components.

## 1. Introduction

Spacecraft requiring accurate three-axis attitude control are all equipped with star sensors to support attitude determination with high accuracy. In recent years, star tracker technology has seen a remarkable evolution. In particular, these sensors have gained significant improvements in their autonomy and capabilities [1–3]. Indeed, modern star sensors are expected to offer new advanced functionalities in addition to the assessed capability of high-precision pointing determination during low angular rate mission phases. The ultimate goal in modern star sensor design is achieving performance, functionality, and reliability levels that allow star sensors to be the only attitude sensor on-board the spacecraft [4]. In particular, the following advanced functionalities can be cited as characterizing modern star sensors:

- to produce high-accuracy, high-reliability attitude angle and rate estimates without external support;
- to operate in a wide range of mission conditions;
- to solve the lost-in-space problem autonomously and in a short time;
- to deliver angular rate information also when attitude determination is not feasible, as during platform de tumbling or slewing.

These functionalities should be achieved via additional software routines rather than by hardware enhancements (apart from improved sensitivity of photodetectors), and different operating modes should control sensor operation. As a result, software for system control and management becomes very complex.

Among the cited advanced functionalities, one of the most demanding, in terms of algorithm and software complexity and sensor operation management, is the determination of the satellite inertial angular velocity during slewing and/or de-tumbling phases. Indeed, many existing satellites execute slewing maneuvers at rates lower than 1°/s, at which the star sensor is still able to acquire star field images, so that star centroids can be computed on the focal plane. Instead, higher angular rates (>1°/s) are being proposed for high-agility small satellites and next generation Earth Observation satellites [5]; in this case the stars are typically acquired as strips, thus calling for different algorithms to be used for angular rate computations.

On the other hand, there is a growing interest in systems able to propagate attitude of very small satellites (such as CubeSats) using low cost sensors and optics [6] (no star trackers available), in order to maintain accurate attitude estimates during eclipse avoiding the drift that characterizes gyroscopes.

In this paper a technique for angular rate determination based on optical flow computation is analyzed. Besides being adopted for vision-based guidance and control of Unmanned Aircraft Systems, optical flow techniques have found usage in space applications within the fields of remote sensing and space exploration. Regarding spaceborne remote sensing, optical flow measurements have been used for example to estimate glacier motion from multi-temporal sequences of electro-optical (EO) images [7], to detect sandstorms [8], to estimate atmospheric motion from geostationary meteorological satellites [9]. Within space exploration, optical flow approaches have been widely proposed for planetary landing (see for example [10,11]).

The optical flow technique proposed in the paper relies on the computation of a displacement field between successive star images, then a least squares method is used to find the best estimate of the angular velocity vector components in the rotating frame matching the observed displacement field. The effectiveness of the proposed techniques is tested by using star field scenes reproduced by an indoor testing facility and acquired by a commercial-off-the shelf camera sensor, shortly described in the paper. Specifically, star field scenes relevant to representative satellite slewing maneuvers are simulated. Then the corresponding images are processed with the optical flow algorithm in order to extract the angular rate information. This information is then compared with the one used in input to the testing facility.

Satellite angular rates estimation, independent of star identification and attitude measurement, has been also discussed in [12] and more recently in [6,13,14].

In particular, [13] discusses a technique that (unlike the one presented in this work) is applicable to electronic rolling shutter imaging mechanisms, since it is aimed at compensating distortion effects due to this technology, thus improving centroiding accuracy and attitude measurement performance in nominal conditions.

In [6] the q-method [15] is used to solve the relative attitude problem between successive frames, while [12] refers to Poisson relation as the basic algorithm equation. [14] illustrates an angular velocity technique based on a least squares approach that starts from knowledge of star vectors and the time sampling interval, and focuses on dynamic estimation techniques such as adaptive Kalman filtering. Validation is based on numerical simulations and night sky observations.

With regards to these latter works, the work presented in this paper provides the following original contributions:

- the entire angular velocity measurement process is presented comprising accurate and efficient optical flow computation and relation with algorithm tuning;
- a complete theoretical error budget is developed that allows predicting the expected measurement accuracy as a function of camera and geometric parameters;
- the developed methodology is tested in hardware-in-the-loop simulations of representative satellite slewing maneuvers.

The paper is organized as follows: Section 2 describes the adopted algorithm with a preliminary error budget to estimate the expected angular accuracy, then Sections 3 and 4 describe, respectively, the adopted indoor facility and the simulation scenario, and the results of the algorithm test on star field scenes acquired with the laboratory facility.

## 2. Algorithm

The developed algorithm is composed of a few basic steps: given a couple of subsequent star field images, first the acquired images are pre-processed to eliminate background noise, and the velocity vector field (which is indeed a displacement field) is calculated in pixels. Then, unit vectors and unit vector derivatives corresponding to the computed velocity vectors are evaluated by exploiting a neural network calibration to estimate at the same time intrinsic and extrinsic parameters relevant to the adopted experimental setup. Once unit vectors and their derivatives are known, the Poisson's equation expressing the time derivative of a unit vector in a rotating reference frame and a least square method are used to find the best estimate of the angular velocity vector components in the rotating frame.

**Figure 1.**
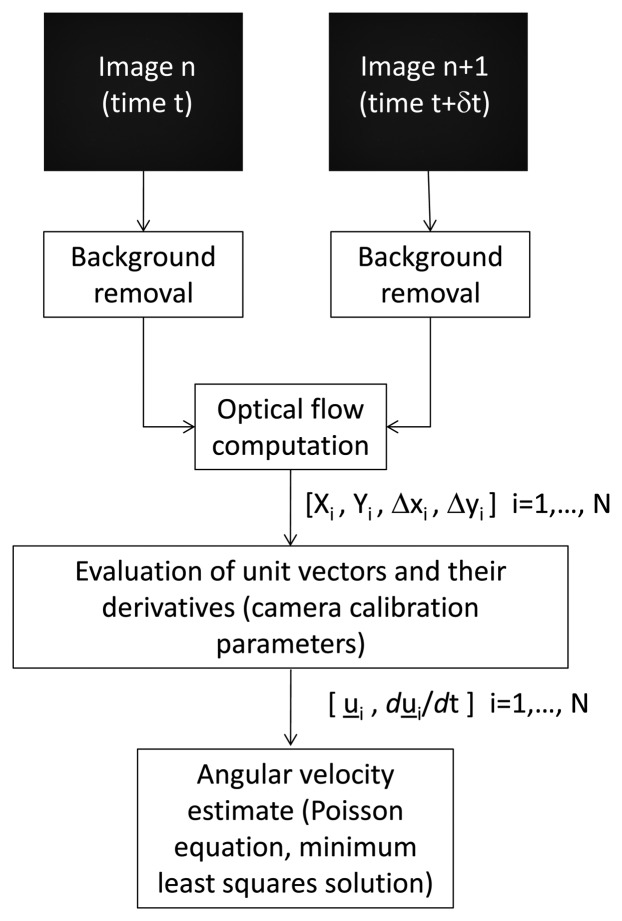
Algorithm flow-chart.

The above mentioned process is summarized in Figure 1. The different blocks are described in details in the following sub-sections, with particular regard to the adopted optical flow methodologies and the equations used for estimating the angular velocity.

### 2.1. Image Processing and Optical Flow Computation

Given a couple of consecutive grey level images, first of all a background noise removal process is carried out separately on both images to eliminate sensor noise which can affect accuracy of optical flow computation. To this end, a global threshold technique [16] is applied in which a μ + 3σ threshold is applied to identify the illuminated pixels, with μ and σ being, respectively, the intensity mean and standard deviation computed over the entire image. All the pixels with intensity below the noise threshold are set equal to zero. This processing may slightly affect centroid accuracy in dynamic conditions when stars are spread over several pixels and the signal-to-noise ratio is degraded, as it will be discussed in the following when dealing with results from high rate simulations.

**Figure 2.**
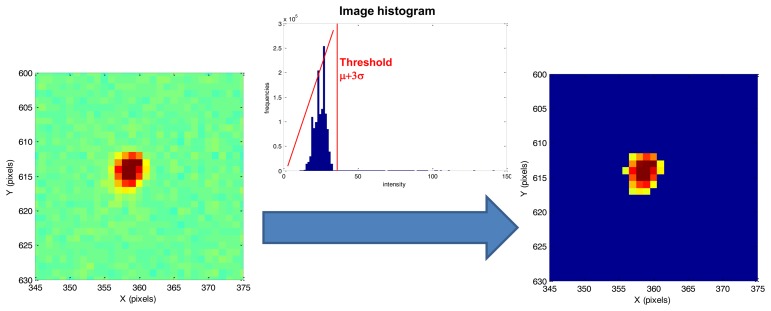
Background noise removal process (pseudo colors are used for the sake of clarity).

An example of background noise removal process around a star is reported in Figure 2. After background noise removal, a labeling technique [16] is applied to distinguish the different stars detected on the focal plane. Within this phase, stars whose dimension is smaller than three pixels are discarded to increase algorithm accuracy, as it is better explained in the error budget sectiosn. It is important to underline that all the subsequent calculations are applied only to the detected stars and not to the whole image. This thresholding procedure significantly reduces the computational burden of optical flow techniques, which is very important in view of real time applications. In fact, modern, multifunction star trackers with large-medium size field of view (FOV, e.g., 15° to 20°) and capable of autonomous multi-mode operation have a detection limit up to visible magnitude m_v_ of 6–6.5. Assuming as reference a 20°-FOV and m_v_ = 6.2 as detection limit, the resulting average number of detectable stars in the sensor FOV is 40 [17].

In general, the optical flow is the 2-D motion field, which is the perspective projection onto the image plane of the true 3-D velocity field of moving surface in space [18,19], arising from the relative motion between the surface and the viewer.

The basic assumption in measuring the image motion is that the intensity structures of local time-varying image regions are approximately constant for, at least, a short time duration. The classical “optical flow constraint equation” [20] can be expressed in differential terms as follows:

(1) $$\frac{\partial I}{\partial x}V_{x}+\frac{\partial I}{\partial y}V_{y}+\frac{\partial I}{\partial t}=0$$

where *I* represents the image intensity, *x* and *y* the two spatial coordinates in the image, *Vx* and *Vy* the corresponding apparent velocity components, and *t* is time.

Different approaches can be adopted to compute optical flow [20–22] such as differential techniques, phase-based and energy based methods, and region-based matching.

Differential techniques compute velocity from spatiotemporal derivatives of image intensity or filtered version of the images (using low pass or band pass filters). In this framework, Equation (1) is an under-constrained equation, since only the motion component in the direction of the local gradient of the image intensity function may be estimated: this is known as “the aperture problem” [20] and one more assumption is necessary.

As an example, Horn and Schunck's method assumes that the motion field is smooth over the entire image domain and tries to maximize a global smoothness term [20], while Lucas and Kanade's method (first introduced in [22] and then developed into the most implemented tracking algorithms [23–25]) divides the original image into smaller sections, assumes a constant velocity in each section, and performs a weighted least-square fit of the optical flow constraint equation, to a constant model for the velocity field in each section.

Differential techniques are not the best solution in the considered case for several reasons. First of all, after background removal, images are very sparse, with a few non zero pixels and a significant departure from the smoothness properties these techniques are based on. Thus, accurate numerical differentiation is typically unachievable. This also happens if background removal is not applied because of the negative impact of noise. Then, it has to be considered that if a very high resolution camera is used, *i.e.*, with a very small Instantaneous FOV (IFOV, *i.e.*, the angle subtended by a single pixel of the imaging system) as it typically happens for a star tracker, apparent star motion can be of several pixels per frame even during medium-rate rotations, while differential techniques typically work well for apparent velocities of the order of 1 pixel/frame, at most. Coarse to fine pyramid representations can be used [24], but with high computational cost since they should be carried out over the entire image, and with degraded performance because of the very sparse image structure.

Since phase-based and energy-based methods work in the Fourier domain, in the star sensor case they also suffer from the same problems of differential techniques.

Region based matching is, instead, an appealing solution because it works well even in noisy images without smooth intensity patterns, and in case of large pixel velocities, such as the ones we have to work with.

The basic principle is to evaluate velocity as the displacement that yields the best fit between image regions at different times. Specifically, in the considered application, a customized two-step method is adopted in which a coarse estimate of the star displacement on the focal plane is computed first and then refined to improve accuracy in the velocity field estimate:

- First of all, the integer shift in pixels (*d*) is computed for each star that minimizes over *δ* the sum of squared differences:
  (2) $$\text{SSD}(x,y,\underline{\delta })=\sum_{j=-k}^{k}\sum_{i=-k}^{k}{\left[I_{n}(x+i,y+j)-I_{n+1}(x+i,\delta_{x},y+j+\delta_{y})\right]}^{2}$$
  As before, *I_n_* and *I_n_*_+_*_1_* indicate two consecutive star images. The sum is calculated on a window whose center is the star centroid calculated in the first image (whose coordinates are *x* and *y*) and whose dimensions (*i.e.*, *k*) depend on the maximum foreseen star dimensions, while *δ* has to vary in an interval which depends on the maximum measurable star displacement. These are the basic parameters for algorithm tuning, and the computational burden of the algorithm increases for larger angular velocities to be measured;
- The coarse estimate of *d* is then refined by computing in the second image the centroid of a window centered at the coarse estimation, whose size and shape are the same of the considered star, plus a margin of 2 pixels. This margin is used to ensure that all the pixels of the considered star (whose intensity is above the threshold) are used for centroid computation in the second image. In fact, the coarse centroid computation has an intrinsic accuracy of 1 pixel due to the integer nature of the solution, and one more pixel is considered as a “safety margin”. This second step is customized to the considered application. It allows a very precise determination of *d* with a very small increase of the computational weight, as it needs very few pixels to be further processed.

The two steps are repeated for each star detected and labeled in the first image. Once star displacements are determined, the information can be easily translated in a velocity information (in pixels) by taking the frame rate into account. Within this framework, it is assumed that accurate image timing is available, thanks to the adoption of proper hardware (camera and shutter technique) and software (real time operating systems and proper coding of image acquisition).

### 2.2. Angular Velocity Estimation

Once star centroids and vector displacements between two consecutive frames are known, the subsequent step is to convert this information in unit vectors and their derivatives. This has to take camera calibration parameters into account and can be done in different ways.

For example, a classical calibration procedure can be used to estimate, firstly, camera intrinsic parameters to be used in a pinhole camera model plus distortion effects (e.g., focal length, optical center, radial and tangential distortion, *etc.*) [16,26], and, then, the extrinsic parameters relevant to the test facility (*i.e.*, the translation vector from camera optical center to a point on the LCD screen assumed as the origin of the display reference frame, and the rotation matrix that relates camera reference frame to the axes of the display reference frame).

In the considered case, an end-to-end neural-network-based calibration procedure is used, which correctly takes account of all the intrinsic and extrinsic parameters relevant to the camera and the test facility [27,28].

Once unit vectors and their derivatives are known, angular velocity estimation is based on the Poisson equation, that relates the temporal derivatives of the stars unit vectors in the Inertial Reference Frame (IRF) and in the Star sensor Reference Frame (SRF):

(3) $${\frac{\partial \underline{u}}{\partial t}|}_{\text{IRF}}{=0=\frac{\partial \underline{u}}{\partial t}|}_{\text{SRF}}+\underline{\omega }\wedge \underline{u}$$

where we take into account that stars are fixed in the IRF, *u* is the star unit vector, and *ω* represents the angular velocity of the SRF with respect to the IRF.

Equation (3) can be rewritten through the vectors components in the SRF as:

(4) $$\left[\begin{array}{ccc} 0 & u_{3s} & -u_{2s}\\ -u_{3s} & 0 & u_{1s}\\ u_{2s} & -u_{1s} & 0 \end{array}\right]\;\left[\begin{array}{c} \omega_{1s}\\ \omega_{2s}\\ \omega_{3s} \end{array}\right]=-\left[\begin{array}{c} \frac{\partial u_{1s}}{\partial t}\\ \frac{\partial u_{2s}}{\partial t}\\ \frac{\partial u_{3s}}{\partial t} \end{array}\right]$$

Thus, three non independent linear equations (in three unknown variables) can be written for each star, leading to N × 3 linear equations if N is the number of stars for which the optical flow has been calculated.

These N × 3 equations can be solved in ω by a classical minimum-least-squares technique based on orthogonal-triangular decomposition, which is computationally light thanks to the sparse structure of the problem matrix. Once the solution for ω is obtained, measurement residuals can be calculated to detect anomalous values and thus to have a first assessment of the method reliability.

### 2.3. Performance Analysis

A theoretical analysis can be carried out to derive a first order error budget for the selected technique. The input parameters for the error budget are: the angular resolution of the considered sensor, the angular velocity to be measured and the consequent velocity field pattern of the stars, the attitude of the SRF with respect to the inertial reference frame (which determines the star distribution within the camera field of view), and the number of detected stars (which depends on star sensor sensitivity and, again, on sensor attitude).

Equation (4) can be rewritten as:

(5) $$\left[\begin{array}{c} \omega_{2s}u_{3s}-\omega_{3s}u_{2s}\\ \omega_{3s}u_{1s}-\omega_{1s}u_{3s}\\ \omega_{1s}u_{2s}-\omega_{2s}u_{1s} \end{array}\right]=-\left[\begin{array}{c} \frac{\partial u_{1s}}{\partial t}\\ \frac{\partial u_{2s}}{\partial t}\\ \frac{\partial u_{3s}}{\partial t} \end{array}\right]$$

**Figure 3.**
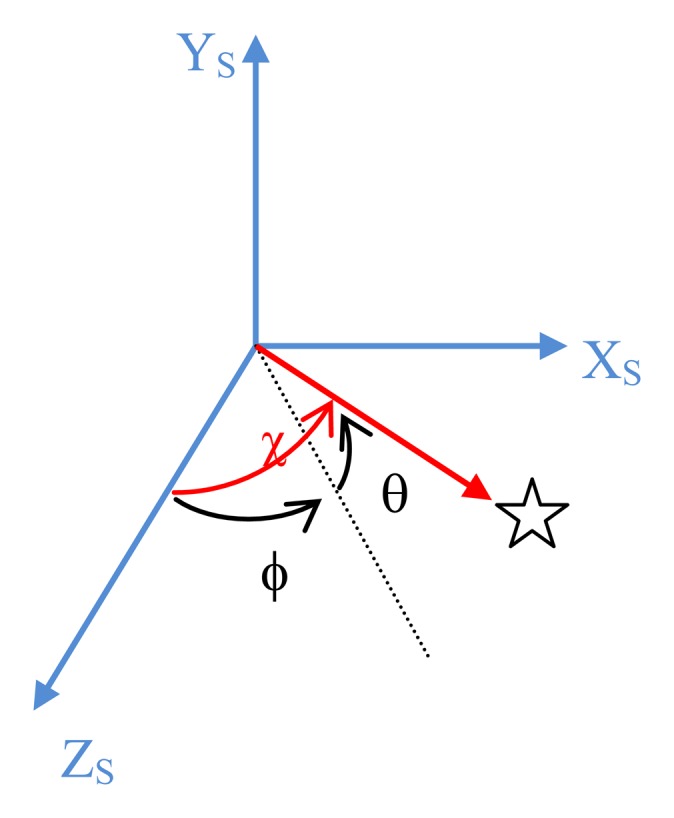
Definition of the generic star angles in SRF: the star line-of-sight is in red, Z_s_ is the sensor boresight axis.

With reference to Figure 3 let us introduce the angles ϕ and θ that define the star line of sight orientation in SRF: θ is the elevation angle over the X_s_,Z_s_ plane of the star line-of sight, and ϕ is the angular separation from the sensor boresight Z_s_ of its projection on X_s_,Z_s_. In addition, we define χ as the angle of the generic star line-of-sight with respect to the sensor boresight axis.

The error analysis can be carried out separately for the different components of the angular velocity in SRF (ω_1s_, ω_2s_, ω_3s_). Let us first consider ω_1s_, *i.e.*, the case under analysis is ω_2s_ = ω_3s_ = 0, ω_1s_ ≠ 0. In this case Equation (5) reduces to:

(6) $$\left[\begin{array}{c} 0\\ -\omega_{1s}u_{3s}\\ \omega_{1s}u_{2s} \end{array}\right]=-\left[\begin{array}{c} \frac{\partial u_{1s}}{\partial t}\\ \frac{\partial u_{2s}}{\partial t}\\ \frac{\partial u_{3s}}{\partial t} \end{array}\right]$$

The unit vector components can be written in terms of the ϕ and θ angles. Since star sensors typically have small FOVs, we can apply the small angle approximation thus getting:

(7) $$\{\begin{array}{c} u_{1s}=\cos\theta \sin\phi \approx \phi \\ u_{2s}=\sin\theta \approx \theta \\ u_{3s}=\cos\theta \cos\phi \approx 1 \end{array}$$

And from Equation (6):

(8) $$\left[\begin{array}{c} 0\\ -\omega_{1s}\\ \omega_{1s}\theta \end{array}\right]≅-\left[\begin{array}{c} \dot{\phi }\\ \dot{\theta }\\ \frac{\partial u_{3s}}{\partial t} \end{array}\right]$$

Then, we can relate the ϕ and θ rate of change directly to the star displacement on the focal plane:

(9) $$\dot{\phi }≅\frac{{\dot{x}}_{c}}{f}$$

(10) $$\dot{\theta }≅\frac{{\dot{y}}_{c}}{f}$$

where *f* is the sensor focal length and *x_c_* and *y_c_* are the coordinates on the focal plane of the generic star centroid.

Thus, we get the final approximate relation in which the first component of the inertial angular velocity vector is directly related to the velocity component along the y_s_ axis computed by means of the optical flow techniques and expressed as an angular velocity:

(11) $$\omega_{1s}≅\dot{\theta }≅\frac{{\dot{y}}_{c}}{f}≅V_{y}$$

Equation (11) allows us to derive the error budget for ω_1s_. In what follows, we use *x* and *y* as non-dimensional coordinates, *i.e.*, they are calculated as $x=\frac{x_{c}}{f}$ and $y=\frac{y_{c}}{f}$.

From a numerical point of view:

(12) $$V_{y}≅\frac{y_{n+1}-y_{n}}{\Delta t}$$

where *n* and *n* + *1* refer to two generic successive frames, Δ*t* is the time elapsed which is inversely proportional to sensor frame rate. Thus we have, for a single star:

(13) $$\sigma_{V_{y}}≅\frac{\sqrt{2}}{\Delta t}\sigma_{y}≅\frac{\sqrt{2}}{\Delta t}\cdot \left(\frac{\text{IFOV}}{\sqrt{N_{\text{starpixels}}}}\right)$$

where *N_starpixels_* is the number of pixels of the focal plane collecting the radiation from the generic star. The term between brackets in Equation (13) approximates the actual accuracy of the centroiding operation.

Since ω_1s_ represents a rotation around an axis perpendicular to the sensor boresight, the corresponding velocity field measured on the focal plane is uniform, *i.e.*, it does not depend on the distance from the boresight axis. Thus, if N is the number of detected stars, since the number of pixels of the different stars is more or less the same, we can produce an estimate of ω_1s_ by combining N identical, and identically distributed, measurements of *Vy*. Thus the uncertainty in ω_1s_ does not depend on the star position in the FOV and it can be estimated as:

(14) $$\sigma_{\omega_{1s}}≅\frac{\sigma_{V_{y}}}{\sqrt{N}}≅\frac{\sqrt{2}}{\Delta t}\cdot \frac{\text{IFOV}}{\sqrt{N}\sqrt{N_{\text{starpixels}}}}$$

**Figure 4.**
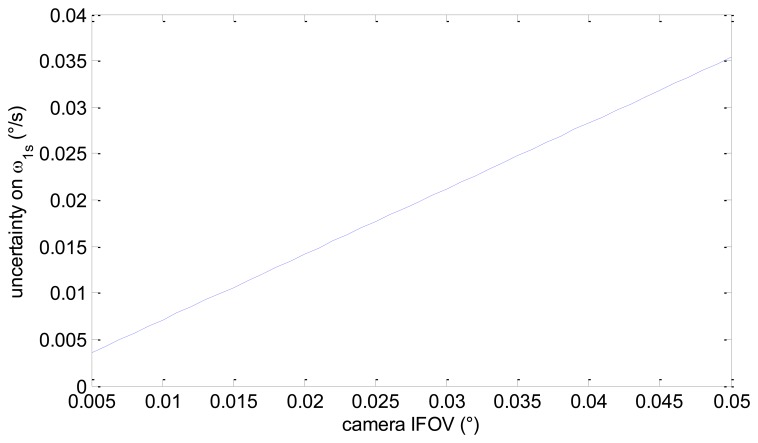
Approximate theoretical uncertainty in ω_1s_ estimate as a function of sensor IFOV.

Assuming realistic values for the frame rate (10 Hz), the number of pixels per star (10), and the number of detected stars (40), we get the uncertainty in ω_1s_ as a function of camera IFOV presented in Figure 4. It can be seen that within the considered range for camera IFOV, the uncertainty in ω_1s_ goes from about 0.0035°/s to about 0.035°/s.

Uncertainty in ω_2s_ can be estimated exactly in the same way, and the error budget is identical since azimuth and elevation IFOVs usually coincide. It is worth noting that the estimated uncertainty does not depend on the angular rotation value which produced the observed velocity field. Of course this conclusion relies on the validity of the proposed model, depending on the assumption that the slew rate is small enough so that a star-field image can be imaged on the focal plane in the considered subsequent images.

The error budget in ω_3s_ is somewhat different. Combining Equations (5) and (7) in the case ω_1s_ = ω_2s_ = 0, ω_3s_ ≠ 0, and with the small angles assumption, we get:

(15) $$-\omega_{3s}\theta ≅-\frac{\partial u_{1s}}{\partial t}$$

(16) $$\omega_{3s}\phi ≅-\frac{\partial u_{2s}}{\partial t}$$

(17) $$\frac{\partial u_{3s}}{\partial t}≅0$$

Combining Equations (15) and (16), and taking Equations (7) into account, we get:

(18) $$\omega_{3s}\sqrt{\phi^{2}+\theta^{2}}≅\sqrt{{\dot{\theta }}^{2}+{\dot{\phi }}^{2}}$$

The first term can be further developed by using spherical trigonometry. Indeed, with reference to Figure 3 we have:

(19) cosχ=cosϕcosθ

where χ is the angle between the direction to the generic star and the sensor boresight axis. Then:

(20) $$\sin^{2}\chi =\sin^{2}\theta +\sin^{2}\phi -\sin^{2}\phi \sin^{2}\theta$$

From the small angle assumption we get:

(21) $$\chi^{2}≅\theta^{2}+\phi^{2}$$

Thus, from Equation (18) we get:

(22) $$\omega_{3s}\chi ≅\sqrt{{\dot{\theta }}^{2}+{\dot{\phi }}^{2}}$$

The χ angle obviously depends on the observed star, and its maximum value depends on the FOV size.

Equation (18) can be rewritten by using the non-dimensional coordinates *x* and *y* as follows:

(23) $$\omega_{3s}≅\frac{\sqrt{{V_{x}}^{2}+{V_{y}}^{2}}}{\sqrt{x^{2}+y^{2}}}$$

and the uncertainty in ω_3s_ can be then calculated at a first order, and for a single star, as:

(24) $$\sigma^{2}\omega_{3s}≅{\left(\frac{\partial \omega_{3s}}{\partial x}\right)}^{2}\sigma^{2}x+{\left(\frac{\partial \omega_{3s}}{\partial y}\right)}^{2}\sigma^{2}y+{\left(\frac{\partial \omega_{3s}}{\partial V_{x}}\right)}^{2}\sigma^{2}V_{x}+{\left(\frac{\partial \omega_{3s}}{\partial V_{y}}\right)}^{2}\sigma^{2}V_{y}$$

By developing the different terms we get:

(25) $$\begin{array}{l} \sigma^{2}\omega_{3s}≅{\left(-\frac{xV}{\chi^{3}}\right)}^{2}\sigma^{2}x+{\left(-\frac{yV}{\chi^{3}}\right)}^{2}\sigma^{2}y+{\left(\frac{V_{x}}{V_{\chi }}\right)}^{2}\sigma^{2}V_{x}+{\left(\frac{V_{y}}{V_{\chi }}\right)}^{2}\sigma^{2}V_{y}≅\\ ≅{\left(-\frac{V}{\chi^{2}}\right)}^{2}\sigma^{2}x+{\left(\frac{1}{\chi }\right)}^{2}\sigma^{2}V_{x} \end{array}$$

where:

$$V=\sqrt{V_{x}^{2}+V_{y}^{2}}$$

and it has been assumed that:

$$\begin{array}{c} \sigma^{2}x=\sigma^{2}y\\ \sigma^{2}V_{x}=\sigma^{2}V_{y} \end{array}$$

By using Equations (12) and (13) we finally have:

(26) $$\sigma^{2}\omega_{3s}≅{\left(-\frac{V}{\chi^{2}}\right)}^{2}\frac{\text{IFO}V^{2}}{N_{\text{starpixels}}}+{\left(\frac{1}{\chi }\right)}^{2}\frac{2\cdot \text{IFO}V^{2}}{\Delta t^{2}N_{\text{starpixels}}}={\left(-\frac{\omega_{3s}}{\chi }\right)}^{2}\frac{IFOV^{2}}{N_{\text{starpixels}}}+{\left(\frac{1}{\chi }\right)}^{2}\frac{2\cdot IFOV^{2}}{\Delta t^{2}N_{\text{starpixels}}}$$

For the typically encountered angular velocities and high frame rates (10 Hz or more), the second term in the above equation is larger than the first one, which yields the following approximate form of the uncertainty in ω_3s_ for a single star:

(27) $$\sigma \omega_{3s}≅\left(\frac{1}{\chi }\right)\frac{\sqrt{2\cdot }\text{IFOV}}{\Delta t\sqrt{N_{\text{starpixels}}}}=\left(\frac{1}{\chi }\right)\sigma V_{x}$$

Equation (27) shows the very intuitive result that the uncertainty in the estimation of the apparent velocity affects the estimation of angular velocity in a way which depends on the star position in the field-of-view: the farther the star line-of-sight is from the boresight, the more accurate the angular velocity estimate will be for a given optical flow uncertainty.

The final ω_3s_ estimate is obtained by combining star measurements having different error distribution. However, a preliminary estimate of the ω_3s_ uncertainty can be obtained by taking an average value of χ and using again the factor $\frac{1}{\sqrt{N}}$.Thus we get:

(28) $$\sigma_{\omega_{3s}}≅\left(\frac{1}{\chi }\right)\frac{\sqrt{2\cdot }\text{IFOV}}{\Delta t\sqrt{N}\sqrt{N_{\text{starpixels}}}}=\left(\frac{1}{\chi }\right)\sigma_{\omega_{1s}}$$

**Figure 5.**
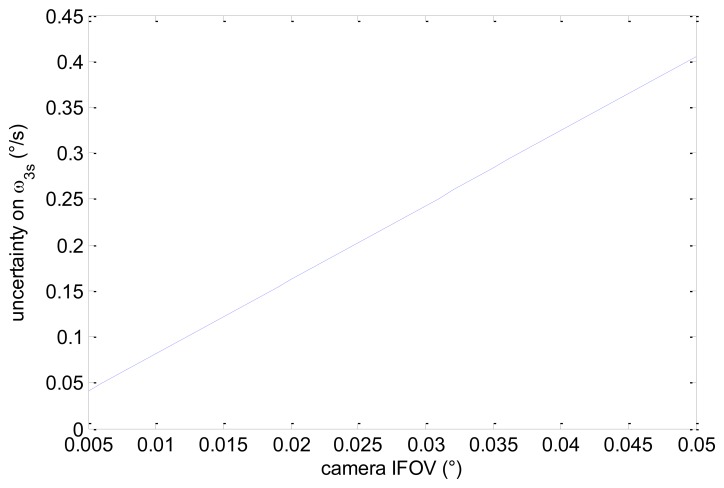
Theoretical uncertainty in ω_3s_ as a function of sensor IFOV.

Considering an average value of 5° for χ (realistic considering typical medium-large size FOVs) we get that the achievable accuracy is about one order of magnitude worse than the one attainable for ω_1s_. This is also consistent with the usual difference existing between the attitude measurement uncertainties across and along the boresight axis of a star sensor [17]. Assuming again a frame rate of 10 Hz, an average number of 10 pixels per star, and 40 detected stars, in Figure 5 we get the uncertainty in ω_3s_ as a function of camera IFOV. Of course, the actual estimation uncertainty depends on the distribution of detected stars within the sensor FOV, and thus also on the actual attitude of the satellite.

## 3. Hardware-in-the-Loop Facility

Tests for performance assessment of the discussed procedure were carried out by means of a functional, hardware prototype of star sensor operated in a laboratory facility for star field scene simulation.

The star sensor prototype was designed to implement the operational modes suggested by the European Space Agency [29]: autonomous operation, initial acquisition from lost-in-space state, attitude tracking, cartography mode for in-depth operation monitoring. It was realized by using COTS hardware: MATROX IRIS P1200HR System [30] is the hardware basis while sensor algorithms were developed in-house. The IRIS P1200HR is composed of two separate units: camera head and a compact embedded CPU which makes this camera fully programmable (it is a so called “smart sensor”). The former exploits SONY CCD detector and focal plane electronics, the latter is based on a 400-MHz Intel Celeron processor equipped with 128-MB RAM, 128-MB flash disk, Microsoft Windows CE 5.0 operating system. Camera head is connected to the processor unit by means of a standard Camera Link™ cabling. Main sensor specifications are in Table 1. Sensor algorithms and the relevant performance are discussed in the literature [31,32].

**Figure 6.**
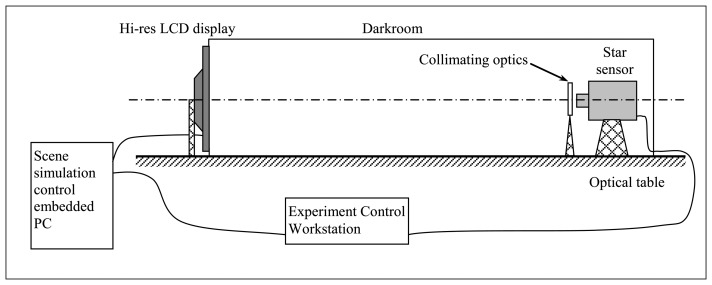
Laboratory facility set-up for star field simulation and star sensor tests.

The laboratory test facility (Figure 6) consists of a dark room where a high-resolution, computer-controlled LCD display produces star field scenes as computed on the basis of a star catalog and of assigned star sensor orientation [27,28]:

- a single pixel of the LCD screen is exploited to simulate a single star of a star field if a static pointing is considered or in the case of a low-rate dynamics of the orbiting platform. Differently, when high-rate attitude dynamics are accounted for in the simulation, a single star is represented by the strip of pixels reproducing its apparent trajectory in the sensor FOV during the update time of the displayed star field scene. Pixel brightness control is used to reproduce star apparent brightness. Approximations result in this simulation approach as a consequence of spatial, temporal, and pixel brightness digital discretization of the synthetic star field scenes and relevant sequences. However a theoretical, worst-case analysis [27] showed that, for high rate dynamic rotation simulation, approximation on large velocity components is at most of the order of 0.01°/s, taking into account the typical number of simulated stars. As it is shown in the following, this does not represent a significant artificial contribution to the estimated algorithm accuracy;
- a collimating lens allows for simulating the large distance of the star sensor from light source;
- a high-performance video processor is adopted for LCD display control by an embedded computer, to carry out static but also dynamical simulations. The former ones simply consist of sequences of star field scenes, as resulting from assigned sensor attitude. The latter ones reproduce the evolution of the star field observed by the sensor during assigned maneuvers (orbit and/or attitude dynamics), with accurate timing;
- sensor position within the darkroom and collimating lens selection guarantee matching of instrument FOV and LCD apparent angular size. Micro translators and rotators are used for fine regulation and alignment of sensor orientation and facility intrinsic reference frame, *i.e.*, the display;
- finally, precise matching is software-based. In particular, it is realized by means of a neural calibration function used to compensate for residual misalignment after installation in the darkroom, and to adjust sensor output to LCD star angular position finely [27,28,33]. This neural network is trained on the basis of a preliminary set of acquisitions to obtain accordance between input star field and sensor position measurements.

The above hardware is completed by the Experiment-Control Workstation that coordinates simulation and sensor operation during test, and it also generates the needed simulated star field data, off-line before star sensor testing.

Table 2 shows the main feature of the system when it is specialized to be coupled to the star sensor prototype in use.

## 4. Simulation Results

Accuracy and reliability of the proposed method can be evaluated by exploiting the described hardware-in-the-loop facility. In all the simulated cases, a circular equatorial Low Earth Orbit (LEO) at altitude of 500 km is considered. This choice does not compromise the general validity of the results since a wide range of attitude maneuvers is simulated to evaluate the effect of different star image patterns on method accuracy. Initially, the satellite body reference frame (BRF) is supposed to coincide with the classically defined orbital reference frame (ORF), *i.e.*, the axis 1 is along the orbital velocity direction, the axis 2 is anti-parallel to the orbital angular momentum vector, and the axis 3 is in the nadir direction. In all the considered cases, the SRF also initially coincides with the BRF apart from sign conventions. In fact, the axis Ys coincides with the axis 2, whereas the other two axes have opposite directions.

**Figure 7.**
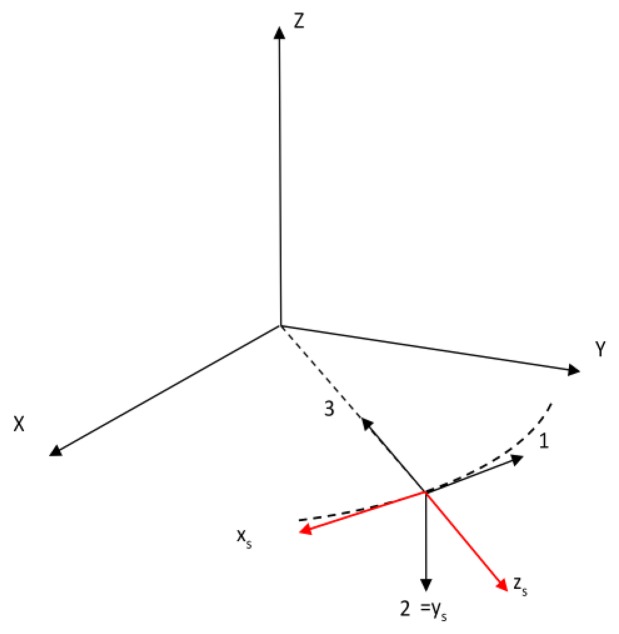
Reference frames for the considered simulations.

The SRF is thus obtained from the BRF by a 180°-rotation around the axis 1. As a consequence, the star sensor boresight axis initially points in zenith direction in the equatorial plane. The reference frames used for the simulations are depicted in Figure 7, with IRF origin at the Earth's centre.

The simulated cases differ for the considered attitude maneuvers. In the first two cases (case 1 and case 2) a satellite rotation around the 1 axis with constant angular velocity (1 deg/s in case 1, 5 deg/s in case 2) is superimposed to the constant angular velocity of the keplerian orbit (6.243·10^−2^ deg/s along the negative 2 axis initially) so that the star sensor boresight axis moves outside the equatorial plane towards the North pole while the satellite rotates around Earth. This condition allows evaluating method performance with a varying number of detected stars and an almost uniform apparent velocity field on the focal plane (pure translation).

In the other two cases (case 3 and case 4), the satellite is supposed to rotate around the star sensor boresight axis, again with constant angular velocity (1 deg/s in case 3, 5 deg/s in case 4). This condition is representative of the case in which the velocity field on the focal plane is not uniform (pure rotation). Actually, a small translational component due to the orbital angular velocity is present in the acquired images.

The simulated angular rates are relevant to the slew maneuvers of many existing satellites, which are typically executed at rates lower than 1°/s. In this condition, the star sensor is able to acquire star field images, and star centroids can be computed on the focal plane. Higher angular rates (>1°/s) are instead proposed for high-agility, small satellites, and next generation Earth Observation satellites [4]. In this case, the stars are typically acquired as strips. This condition can affect the accuracy of the proposed technique.

For reader convenience, all the simulated cases are summarized in Table 3. It is worth recalling that the reported “true” angular velocity components (ω_1s_, ω_2s_, and ω_3s_) represent the components along the SRF axes of the inertial angular velocity vector of the SRF.

### 4.1. Out of Plane Rotation Results

**Figure 8.**
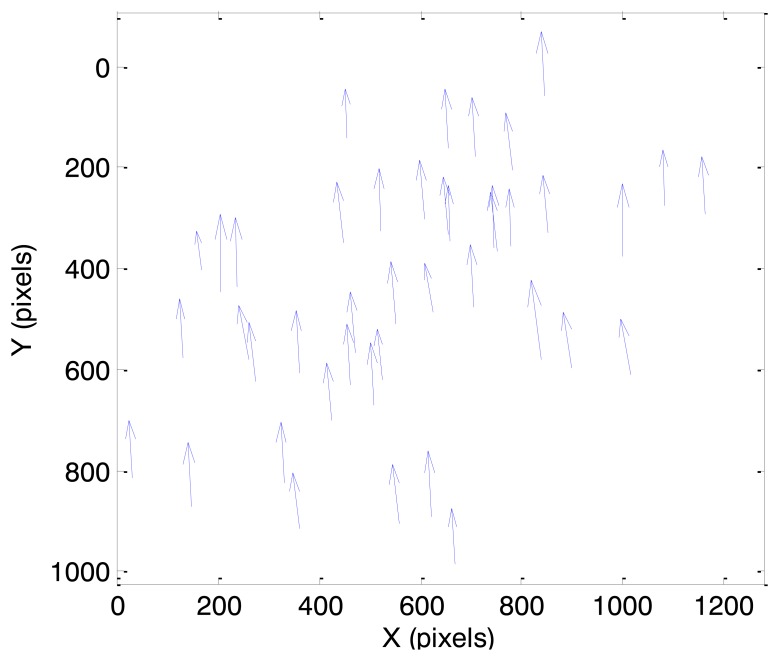
Velocity field as estimated by the optical flow algorithm from a couple of consecutive images (case 1).

In this case, initially the true angular velocity vector has non-zero components only along the x_s_ and y_s_ axes of SRF. As a consequence, the velocity field pattern represents a pure translation with a larger components along the y_s_ axis. This condition is evident in Figure 8, where the velocity vectors calculated from a couple of consecutive frames in case 1 are depicted (magnified for the sake of clarity). In spite of some noise affecting more the (smaller) horizontal velocity component, the uniformity of the velocity field can be clearly appreciated. In the considered case, pixel displacements are of the order of 0.4 pixels for the horizontal component and 5.9 pixels for the vertical component.

**Figure 9.**
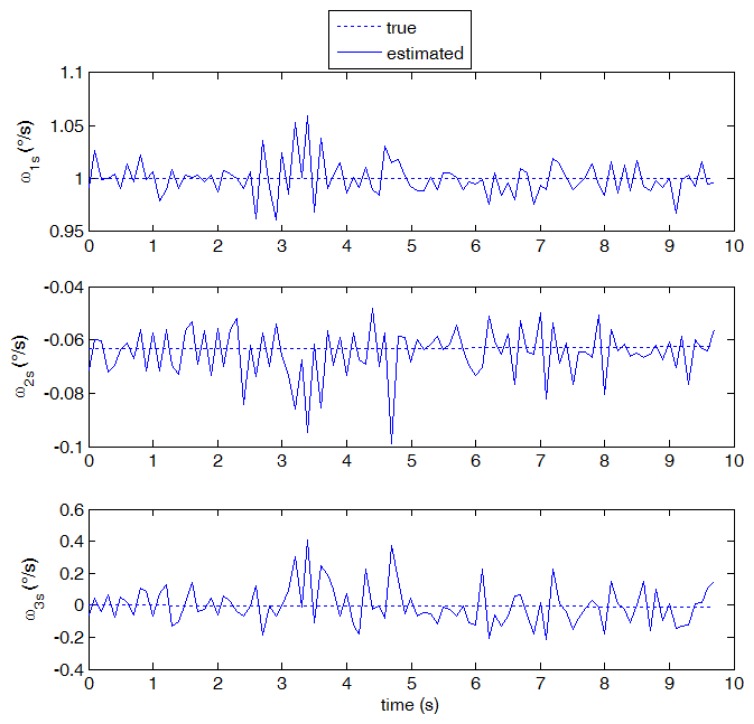
Estimated angular velocity components against “true” values (case 1, 10 frames per second).

As a result of the relatively large number of detected stars, and velocity vectors, both the larger x_s_ component (1 deg/s) and the smaller y_s_ component (0.06 deg/s) are measured with good accuracy, as shown in Figure 9. The described algorithm was run on a sequence of about 100 images, corresponding to a simulation time span of about 10 s. It can be seen that the measurements are unbiased on average, and the measurement noise is very small. The third component estimate is also unbiased, but, in accordance with the error budget analysis, a larger noise is observed in this solution. Slight variations of the number of detected stars (due to stars moving inside or outside camera FOV) are the main cause of small oscillations of measurement noise.

**Figure 10.**
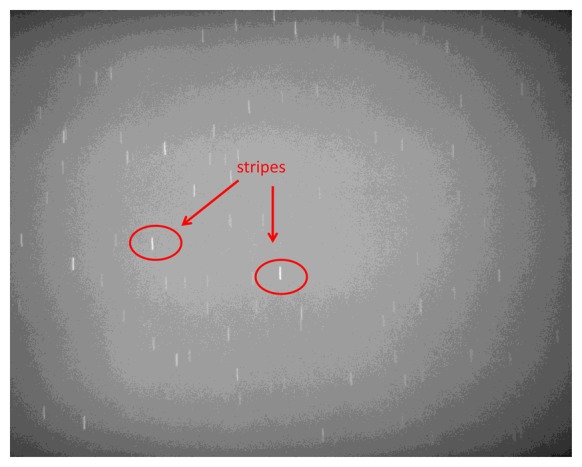
Sample image of star stripes relevant to case 2, significantly modified for the sake of clarity (large angular velocity).

**Figure 11.**
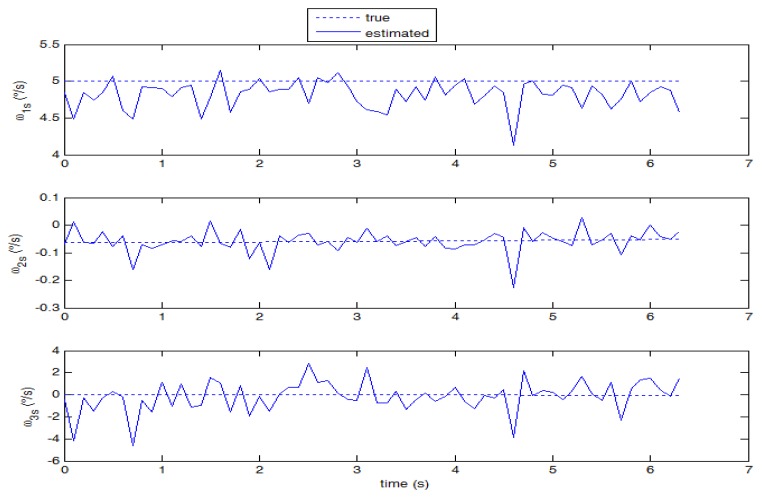
Estimated angular velocity components against “true” values (case 2, 10 frames per second).

Although the proposed technique is specifically tuned to work with star images, it is of great interest investigating its application to cases with higher angular velocities, where stripes rather than stars are imaged on the focal plane and a large displacement in pixels is measured among consecutive frames. Case 2 is representative of this condition (see Figure 10, where the original star image has been significantly modified in brightness and contrast to enhance clarity). In this case, the computational load of the proposed technique increases since large windows have to be used for effective region-based matching. Moreover, the signal to noise ratio in each frame is reduced, thus reducing the number of valid star measurements, and degrading accuracy in estimating star centroids and their displacement. As it is derived from the theoretical error budget, these phenomena increase the uncertainty in the angular velocity estimates. Nevertheless, as shown in Figure 11, the average performance is still satisfying, with the smaller component ω_0_ measured with slightly worse accuracy compared with case 1. Instead, the estimate of ω_1s_ shows a small negative bias (due to a slight under-estimation of stars displacement) and a larger error standard deviation, which is also found in the third component estimate.

### 4.2. Radial Rotation Results

**Figure 12.**
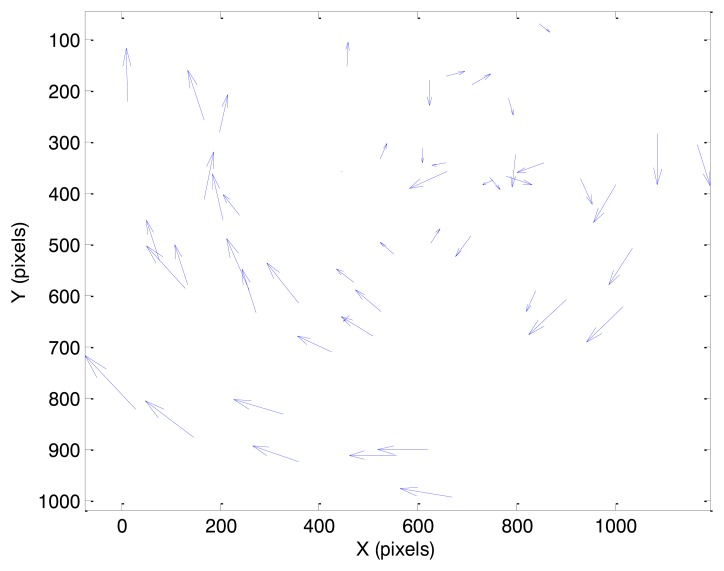
Vector field as estimated by the optical flow algorithm from a couple of consecutive images (case 3).

**Figure 13.**
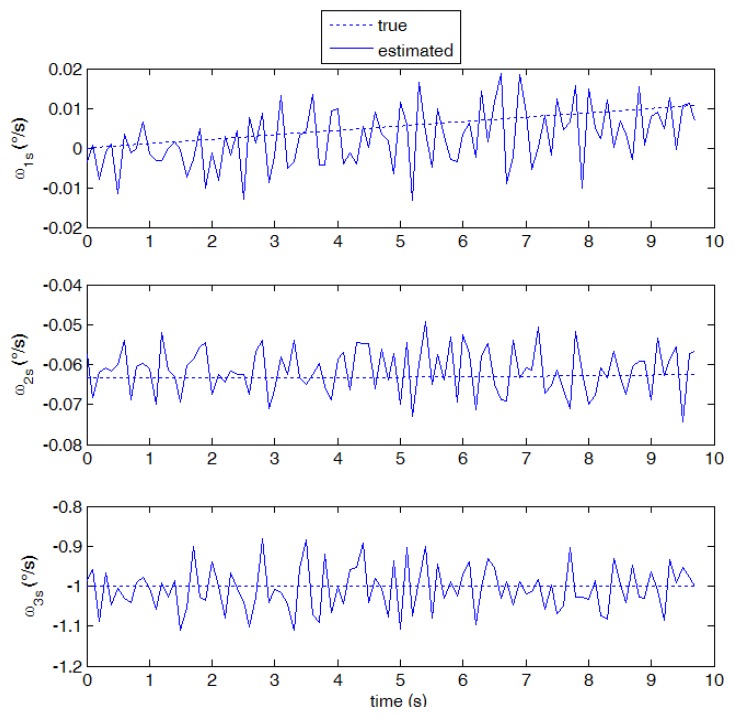
Estimated angular velocity components against “true” values (case 3, 10 frames per second).

**Figure 14.**
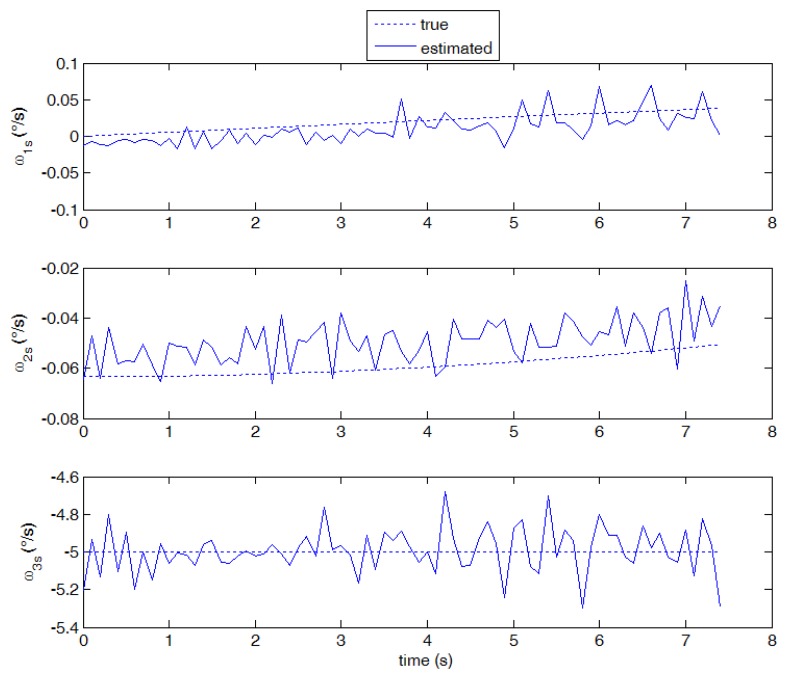
Estimated angular velocity against “true values” (case 4, 10 frames per second).

Considering now the first radial rotation case (case 3), the velocity field pattern is of course very different from the one detected in cases 1 and 2, with a rotation around the boresight axis superimposed to the horizontal translation due to the orbital angular velocity. Notwithstanding the large variation of the velocity modules on the focal plane, the optical flow is able to capture the motion field (shown in Figure 12) and to measure the angular velocity components with good accuracy (see Figure 13). Again, as foreseen by the error budget analysis, a larger noise is found in the estimate of the third velocity component. In the high rotation case (case 4) satisfying performance is maintained and it is in any case better than case 2 in all velocity components (see Figure 14).

Performance in terms of mean and standard deviation of errors with respect to assigned values is summarized in Tables 4 and 5. Specifically, Table 4 shows statistics relevant low slew rates (cases 1 and 3): as foreseen by the error budget analysis, in the out-of-plane case, the standard deviation in ω_1s_ is of order of 10^−2^ deg/s (about 1% of the “true” value), whereas the noise in the boresight axis component is always about one order of magnitude higher. In absolute terms, a slightly better performance is measured in the radial rotation case, which is still in agreement with the theoretical error budget taking into account that the number of detected stars, and the average off-boresight angle (of the order of 55 and 7°, respectively) were larger than the reference values assumed in deriving Figure 4 and 5.

Table 5 shows statistics relevant to case 2 and case 4, which, as previously underlined, represent limiting conditions characterized by high slew rates. Although performance is globally worse, a satisfying accuracy is maintained especially in the radial case. This is mostly due to the fact that strips generated by the fast star movement during sensor acquisition time are shorter in the radial case, as it can be seen from Equations (11) and (23). Since in these high-rate conditions strip length is inversely proportional to the signal-to-noise ratio, this implies a better signal to noise ratio for each star, and thus a larger number of detected stars as well as better accuracy in estimating optical flow between consecutive frames. The standard deviation in third component is always one order of magnitude higher with respect to the first component.

## 5. Conclusions

This paper focused on an optical flow-based technique to estimate spacecraft angular velocity based on successive images of star fields. The main steps of the developed algorithms are image pre-processing for background removal, region-based optical flow computation, and least-squares solution of a linear system obtained expressing the time derivative of a unit vector in a rotating reference frame for each detected star.

Algorithm performance was evaluated on a set of star images generated with different rates and geometries (1°/s and 5°/s out-of-plane or radial rotations) by a hardware-in-the-loop testing facility and acquired by a commercial-off-the shelf camera sensor.

The method showed good performance in terms of accuracy and reliability, and experimental results were consistent with the developed theoretical error budget taking account of star fields and camera parameters. In the case of the out-of-plane rotation at 1°/s, unbiased angular rate estimates were generated and the measurement noise was of the order of 10^−2^ deg/s for the off-boresight components, while the achievable accuracy for the angular velocity component along boresight was of about one order of magnitude worse. A slightly better performance was estimated in the 1°/s radial rotation case due to the number and the average off-boresight angle of detected stars.

Rotation at 5°/s represents a very challenging situation for angular velocity measurement, with star strips on the image plane and a significant reduction of signal-to-noise ratio. Nevertheless, the developed algorithm was able to measure with satisfying accuracy these velocities, especially in the radial rotation case.

Future work is aimed at optimizing algorithm tuning in view of real-time implementation. In fact, the computational burden dramatically depends on settings related to the maximum angular velocity that has to be measured. From this point of view, a feedback control scheme, where the current algorithm settings depend on the latest angular velocity estimate and the measurement residual, seems to be a promising solution. Furthermore, measurement residual can also be used to generate a real-time estimate of measurement covariance, which allows generated output to be effectively integrated in dynamic filtering schemes, possibly also comprising estimates from other sensors.

## Figures and Tables

**Table 1.** Star sensor prototype specifications.

Field Of View	22.48° × 17.02°
Effective Focal Length	16 mm
F-number	1.4
Star Sensitivity	<visible magnitude 7
Image Sensor	½″ CCD Progressive Scan
Image Size	1,280 × 1,024 pixel
Instantaneous Field Of View	0.017° × 0.017°

**Table 2.** Test facility features relevant to sensor FOV match.

Display active area H × V (m)	0.641 × 0.401
Display resolution H × V (pixel)	2,560 × 1,600
Collimating lens focal length (m)	1.3
Collimator diameter (mm)	50
Display apparent angular size (deg)	27.6 (H) × 17.5 (V)
Display pixel apparent angular size at screen centre (deg)	0.011 × 0.011 (H × V)
Overall magnification ratio (with 16-mm-focal sensor optics)	1.23 × 10^−2^

**Table 3.** Summary of simulated test cases: initial conditions.

	**Out of Plane Rotation**		**Radial Rotation**	

	**Case 1**	**Case 2**	**Case 3**	**Case 4**
ω_1S_ (°/s)	1	5	0	0
ω_2S_ (°/s)	−6.243·10^−2^	−6.243·10^−2^	−6.243·10^−2^	−6.243·10^−2^
ω_3S_ (°/s)	0	0	−1	−5

**Table 4.** Synthetic statistics relevant to low slew rates.

	**Out-of-plane (Case 1)**		**Radial (Case 3)**	

	**Mean**	**Std**	**Mean**	**Std**
Error on ω_1s_ (°/s)	−1.20·10^−4^	1.64·10^−2^	−2.88·10^−3^	6.72·10^−3^
Error on ω_2s_ (°/s)	−1.90·10^−3^	9.20·10^−3^	1.61·10^−3^	5.71·10^−3^
Error on ω_3s_ (°/s)	−4.96·10^−3^	1.22·10^−1^	−6.66·10^−3^	5.57·10^−2^

**Table 5.** Synthetic statistics relevant to high slew rates.

	**Out-of-plane (Case 2)**		**Radial (Case 4)**	

	**Mean**	**Std**	**Mean**	**Std**
Error on ω_1s_ (°/s)	−1.79·10^−1^	1.81·10^−1^	−9.57·10^−3^	1.52·10^−2^
Error on ω_2s_ (°/s)	4.26·10^−4^	3.90·10^−2^	9.61·10^−3^	7.47·10^−3^
Error on ω_3s_ (°/s)	−1.28·10^−1^	1.38	7.95·10^−3^	1.21·10^−1^
